# A diffuse rash in a patient after allogeneic hematopoietic stem cell transplant for AML

**DOI:** 10.1002/ccr3.3306

**Published:** 2020-09-24

**Authors:** Asmaa Ferdjallah, Christina L. Boull, Heather Stefanski, Christen L. Ebens

**Affiliations:** ^1^ Division of Hematology and Oncology University of Minnesota Masonic Children’s Hospital Minneapolis Minnesota USA; ^2^ Division of Pediatric Dermatology University of Minnesota Masonic Children’s Hospital Minneapolis Minnesota USA; ^3^ Division of Blood and Marrow Transplantation Department of Pediatrics University of Minnesota Masonic Children’s Hospital Minneapolis Minnesota USA

**Keywords:** dermatology, hematology, oncology, pediatrics and adolescent medicine

## Abstract

Sweet's syndrome associated with relapse of leukemia suggests abnormal neutrophil response to transformation of dysfunctional leukemia blast cells, and hence, relapse should be excluded in similar clinical situation.

## Graphical abstract

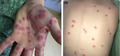

## 1 CASE PRESENTATION

**Figure 1:**
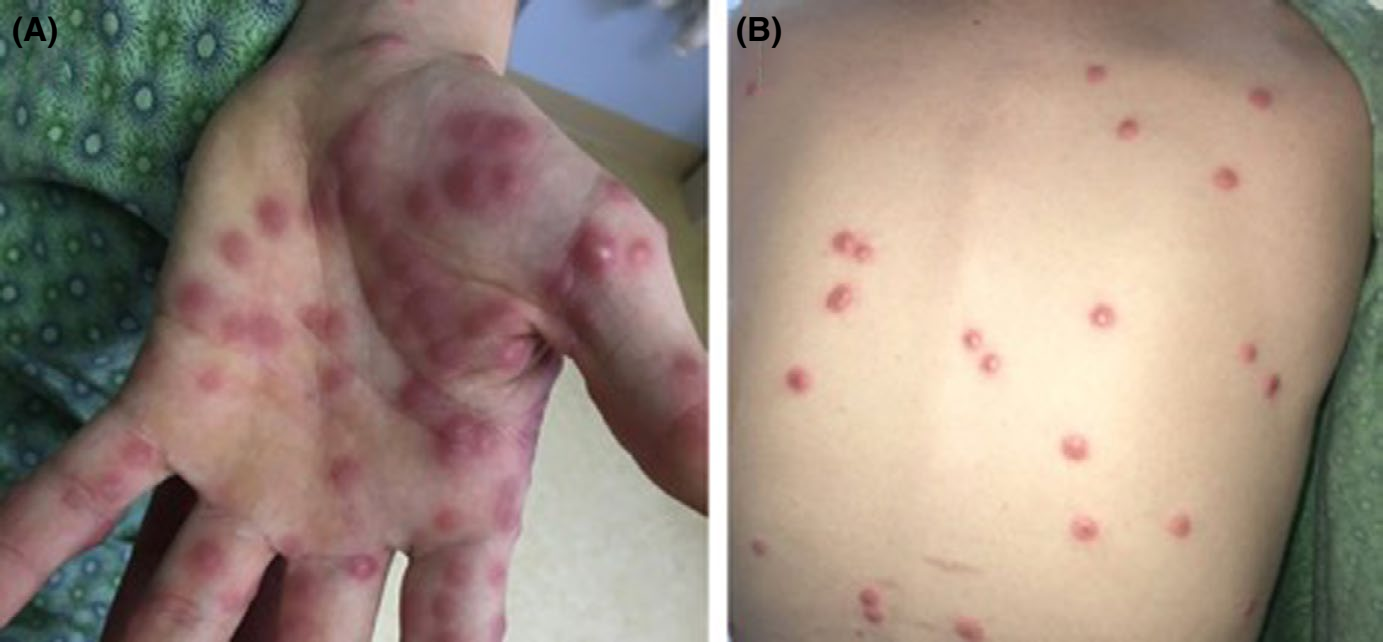
A, Palmar surface of left hand demonstrating diffuse violaceous papules. B, Multiple violaceous papules on the back

**Figure 2:**
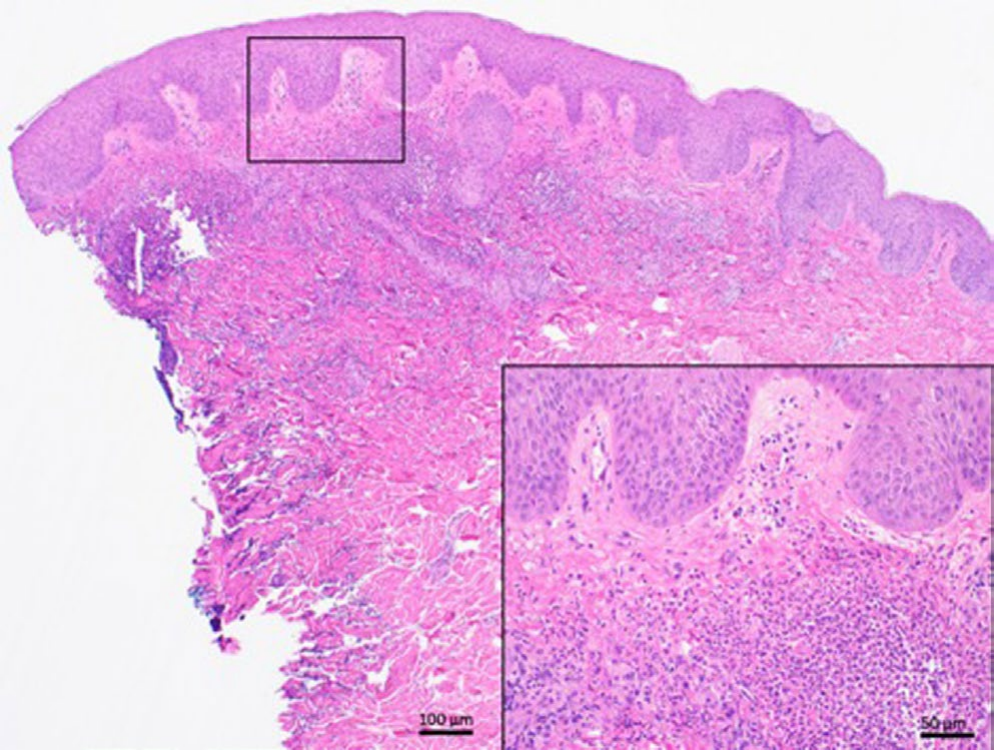
Punch biopsy showing mild epidermal hyperplasia above an inflammatory infiltrate in the upper dermis. H&E staining, 1.25× magnification. Scale bar 100 μm. Insert: Higher power view of many neutrophils without vasculitis. H&E Staining, 40× magnification

A 19‐year‐old male patient presented with a diffuse painful rash 286 days after a single 5/8 HLA‐matched umbilical cord blood transplant (UCBT) for minimally differentiated acute myeloid leukemia (AML). He had achieved neutrophil engraftment on day +17 post‐UCBT, was transfusion independent, and 100% donor engrafted with no evidence of leukemia on his day +180 bone marrow disease evaluations. He had no graft‐versus‐host disease to date and had recently completed immunosuppressant prophylaxis. Two weeks prior to presentation, he was admitted with febrile neutropenia and treated with oseltamivir phosphate for positive influenza B by nasal swab. One week prior to presentation, he noted two “insect bites” on his right arm. Over the course of several days, innumerable tender violaceous papules and vesicles erupted over his entire body, including his palms and soles. Some lesions evolved to develop a hemorrhagic center. He developed lip and oral mucosa lesions, reported pain when swallowing and petechiae were noted on his tongue. He was seen by a local physician who prescribed meloxicam for associated myalgias. During a dermatology appointment for further evaluation at our institution, he was febrile and neutropenic (white blood cell count of 3.8 × 10^9^/L, hemoglobin of 11 g/dL, platelet count of 168 × 10^9^/L, and absolute neutrophil count of 0.5). His electrolytes were within normal limits. A chest x‐ray identified right‐sided nodular densities concerning for infection. These findings prompted admission to the blood and marrow transplant service for further management (Figures 1 and 2).

## 2 WHAT IS YOUR DIAGNOSIS?

- Erythema Multiforme
- Pyoderma Gangrenosum
- Mycoplasma Induced Rash and Mucositis
- Sweet's Syndrome

**Diagnosis**: Sweet's Syndrome.

## 3 DISCUSSION

A 4 mm skin punch biopsy of a lesion on his left upper back confirmed neutrophilic papillary dermal edema consistent with Sweet's syndrome. There was no evidence of vasculitis or malignancy. Classic histopathological findings include a dense diffuse mature neutrophilic infiltrate in the upper dermis, although occasionally in the overlying epidermis or underlying adipose tissue, with edema.^1^, ^2^ Fragmented neutrophil nuclei known as karyorrhexis or leukocytoclasia may be seen.^3^ The lesions are sterile in nature.

Sweet's syndrome is the eponym for acute febrile neutrophilic dermatosis presenting as multiple tender papules involving the extremities and back in an asymmetric distribution.^1^, ^2^ The lesions often have a pale center and can mimic bullae.^4^ Patients present with fever and may appear acutely ill with myalgias.^3^ The syndrome can be triggered by three clinical settings: classical (infection, autoimmune disorder, or pregnancy induced), medication‐induced, or malignancy‐associated.^5^, ^6^, ^7^ Dr Sweet first described the syndrome in the 1960s, but it was not until the 1970s when skin tissue biopsy samples identifying the eponymous syndrome were associated with neoplasm.^3^, ^8^, ^9^

For this patient, peripheral blood flow cytometry revealed increased abnormal blasts (38%) consistent with relapse of his acute myeloid leukemia. Although Sweet's syndrome is a paraneoplastic process associated with the initial diagnosis of myeloid malignancy, it can also present at time of relapse and concurrent with malignancy. In fact, AML is reported as the most common hematologic malignancy temporally associated with Sweet's syndrome.^1^ For this patient population, although Sweet syndrome is secondary to malignancy 20% of the time, it is important to continue to rule out a drug‐induced etiology.

Systemic steroids are considered the gold standard therapy for Sweet's Syndrome with NSAIDs, colchicine, and dapsone reported as less‐effective second line agents.^2^ Given concern for infection on presentation or masking extent of malignancy, providers may be reluctant to proceed with systemic steroids until a definitive diagnosis is made. In malignancy‐associated Sweet's syndrome, treatment of the underlying malignancy is the primary treatment for the dermatologic manifestations of Sweet's syndrome. Lesions typically resolve without evidence of scarring.

### 3.1 Outcome and follow‐up

This patient was started on dexamethasone swish and spit, topical triamcinolone 0.025% ointment, and topical mometasone 0.1% ointment, while diagnostic bone marrow biopsy was preformed, revealing 82% blasts. After confirmation of relapsed acute undifferentiated leukemia, he was started on prednisone 1 mg/kg followed by a slow taper. He demonstrated rapid resolution of his rash and discomfort upon commencement of systemic steroids.

## CONFLICT OF INTEREST

None declared.

## AUTHOR CONTRIBUTIONS

AF: contributed to writing of the paper and literature search. CLB and CLE: contributed to clinical care of the patient, data analysis, critical review, and editing of the paper. CLB: completed medical photography. Written informed consent to publication was obtained.

## ETHICAL APPROVAL STATEMENT

Approval was obtained from the University of Minnesota Institutional Review Committee in accordance with ethical standards.
